# Examining the uptake of COVID-19 vaccine booster doses among healthcare workers in South Africa: A mixed-methods study

**DOI:** 10.1371/journal.pgph.0002639

**Published:** 2023-11-20

**Authors:** Gavin George, Phiwe Nota, Michael Strauss, Emma Lansdell, Remco P. H. Peters, Petra Brysiewicz, Nisha Nadesan-Reddy, Douglas Wassenaar

**Affiliations:** 1 Health Economics and HIV and AIDS Research Division (HEARD), University of KwaZulu-Natal, Durban, South Africa; 2 Division of Social Medicine and Global Health, Lund University, Lund, Sweden; 3 Research Unit, Foundation for Professional Development, East London, South Africa; 4 School of Nursing & Public Health, University of KwaZulu-Natal, Durban, South Africa; 5 South African Research Ethics Training Initiative, School of Applied Human Sciences, University of KwaZulu-Natal, Pietermaritzburg, South Africa; Osun State University, NIGERIA

## Abstract

This study aimed examin the factors associated with the uptake and non-acceptance of COVID-19 vaccine booster doses among healthcare workers (HCWs) in South Africa. We used a mixed-methods design with data from a web-based self-administered survey followed by semi-structured in-depth interviews (IDIs) with selected participants. Of the 6235 HCWs included in our analysis who had fully vaccinated, 3470 (56%) had taken their booster dose with a further 17% intending to get the booster. HCWs aged 35 to 49 years (OR = 1.30 [95% CI: 1.15–1.46]), and those aged 50 years or older (OR = 2.66 [95% CI: 2.32–3.05]) were more likely to get the booster dose. Females were less likely to have received the booster dose (OR = 0.88 [95% CI: 0.79–0.98]) with doctors more likely (OR = 1.58 [95% CI: 1.35–1.84]) than Nurses to have received the booster dose. HCWs in direct contact with patients (OR = 1.17 [95% CI: 1.00–1.38]) and who had previously received a flu vaccine (OR = 1.99 [95% CI: 1.56–2.55]) were more likely to have received the booster dose. Four themes emerged from the qualitative data analysis: *(1) Vaccination as routine practice among HCWs; (2) Emergence of new COVID-19 variants necessitating vaccine boosters; (3) Fear of potential side-effects; and (4) Limited value of COVID-19 vaccine boosters*. Some HCWs broadly accepted the value of vaccination, and believed that boosters were necessary to effectively combat emergent new virus strains, which contrasted with peers who believed that boosters offered little defence against virus mutations. Fear prohibited some HCWs from getting the booster, with some having experienced adverse side effects from their initial vaccination, whilst others were concerned about future complications. Waning booster uptake rates could be arrested through invigorated communication strategies, while effective evidence-based training can potentially create positive normative vaccination practices amongst HCWs.

## Introduction

Healthcare Workers (HCWs) remain susceptible to SARS-CoV-2 infection, making this a priority group for vaccination and access to booster options, as outlined in the WHO’s Strategy to Achieve Global COVID-19 Vaccination by mid-2022 [1]. HCWs in South Africa (SA) were the first group to be afforded the opportunity to get vaccinated (with the Johnson and Johnson (J&J) vaccine through the Sisonke implementation study) from February 2021, with the South African Health Products Regulatory Authority (SAHPRA) in late 2021 approving the use of the Pfizer vaccine as a booster, six months after the administration of the vaccine’s second dose, along with the J&J vaccine as a booster at least two months after primary vaccination [2]. The emergence of new COVID-19 variants of concern, such as Omicron, coincided with the South African government’s renewed calls for the uptake of vaccine boosters [3].

Research among HCWs in SA revealed high initial COVID-19 vaccine uptakes rates [4]. These data reflect global trends, with vaccine uptake rates higher among HCWs when compared to the general population [5, 6]. However, there remains little data on HCWs’ uptake of boosters, with the few studies undertaken suggesting a declining in the uptake of additional vaccine doses [7–9]. This reluctance among HCWs to take booster doses raises concerns given HCWs’ increased risk of contracting and transmitting SARS-CoV-2 in healthcare settings, and the important role they play in building vaccine confidence among the general population, especially as boosters are likely to be crucial to address waning immunity and newly emerging variants [10, 11]. Given this need for booster vaccinations to improve the immunogenicity of the vaccine and prolong protection [10], these sub-optimal booster uptake rates demonstrate the need to understand the determinants of COVID-19 vaccine booster acceptance in HCWs across various settings.

Studies of the acceptance and uptake of COVID-19 vaccine booster doses have focused on HCWs in high-income settings, with little evidence from the African continent and South Africa specifically. This study, therefore, aimed to gain a better understanding of the factors associated with vaccine booster uptake among HCWs in SA. Survey data were augmented by qualitative data revealing HCW perspectives on COVID-19 vaccine booster doses, with the summative evidence providing not only guidance for COVID-19 vaccine booster uptake interventions, but also other routine immunisation programmes.

## Methods

### Ethics statement

The study was granted ethical clearance by the University of KwaZulu-Natal Biomedical Research Ethics Committee (BREC/3970/2022) in compliance with all regulations and policies regarding ethical conduct of research. Written consent was obtained at the beginning of the online survey by providing formal paragraph-wise information about the study, with the participant requiring to click on a button for providing consent on the same online survey platform before moving on to filling the rest of the online survey questionnaire.

### Study design

This study used a mixed-methods design with data from a web-based self-administered survey followed by in-depth interviews (IDIs) with selected participants. Mixed methods research allows for combining elements of qualitative and quantitative research approaches for the broad purposes of providing depth of understanding and corroboration on a particular research topic [12, 13]. Data were collected between August 2022 and October 2022.

### Sampling

The Foundation for Professional Development’s (FPD) database, which comprised contact details of 88 000 HCWs at the commencement of the study, was used to recruit HCWs for this study. Participants were offered compensation in the form of an entry into a draw for one of ten ZAR500 (~US$33) cash vouchers. The draw was not linked to participants’ survey responses. Participants interested in participating in IDIs were asked to provide their contact details following completion of the self-administered survey. A total of 7 763 HCWs participated in the survey. For this paper, we excluded all participants that were either unvaccinated or did not disclose their vaccination status. Thus, final analysis was undertaken on 6 325 vaccinated participants. As part of the survey, participants had the option to indicate their willingness to be contacted for a follow-up interview (IDI). Participants who had provided their contact details were organised into two groups (vaccinated and unvaccinated), then randomly selected using the *randbetween* formula in Microsoft Excel for IDIs and scheduled for a virtual interview. In total we interviewed 30 HCWs, 10 vaccinated and 20 unvaccinated.

### Measures

The survey captured socio-demographic information, COVID-19 history, chronic conditions, and questions on vaccination behaviour. Survey questions were derived from a review of studies evaluating HCW hesitancy towards COVID-19 vaccines, described elsewhere [4].

Two outcome measures were created based on the question; “Are you planning to get a booster vaccination against COVID-19?” with respondents indicating either “I have already received the booster dose”, “yes, as soon as possible”, “yes, but in a few months up to a year”, “yes, but in a year or more”, “I am unsure”, “no, but I might consider it in the future”, and “no, never”. The first outcome measure was coded as a binary variable where: 1 = “I have already received the booster dose” (Received Booster), and 0 = any other response to the question (No Booster). The second outcome measure was coded as a binary variable where: 1 = “I have already received the booster dose” or “yes, as soon as possible” or “yes, but in a few months up to a year” or “yes, but in a year or more” (Received and Intend), and 0 = “I am unsure” or “no, but I might consider it in the future” or “no, never” (Hesitant and Against).

For the IDIs, two interview guides were developed, one for vaccinated HCWs and another for unvaccinated HCWs, with open-ended questions and probes regarding; (1) HCWs’ vaccination behaviour (including vaccine booster doses); (2) HCWs’ experience with administering vaccines; (3) HCWs’ perspectives of the vaccine programme; (4) how HCWs gather and appraise information sources; and (5) HCWs’ perspectives on educational resources that can be used to support them. Interviews were conducted by authors PBN and GG, who are experienced in conducting qualitative interviews using interview guides. IDIs lasted approximately 30 minutes and were recorded and transcribed.

Personal information from study participants is available to the study principal investigator (PI) and co PI’s, this data was transmitted via a password-protected secure web-based file sharing dashboard service. Study personnel also transferred all data to password-protected computers.

### Analysis

For the quantitative data, we ran a series of univariate logistic regression models to determine; how significantly a number of measures influenced a participant’s likelihood of having received a booster compared to those who hadn’t, and how significantly the same measures influenced a participant’s likelihood of having received or intended to receive a booster dose, compared to those who were hesitant or against receiving a booster dose. Finally, we conducted descriptive analysis (frequencies and proportions) of the underlying reasons for hesitancy towards vaccination boosters, stratified by gender.

Qualitative data were analysed thematically using an inductive approach as prescribed by Braun and Clarke [14] six-step process. This process entailed the researchers (PBN and GG) familiarizing themselves with the data by taking notes while conducting the interviews, listening to the recoded interviews, and reading the transcripts. Zoom transcribing software was used to transcribe the recorded interviews. Transcriptions were read and codes developed, after which themes were generated.

In this study, the point of integration was at the data analysis phase where the qualitative data provided in-depth understanding of the quantitative findings, revealing trends on HCWs vaccination attitudes and behaviours. The quantitative and qualitative data were kept analytically distinct where a statistical technique was used to analyse the survey data while thematic analysis was used to analyse interview data, as explained by Tariq and Woodman [15]. Findings from the survey and IDIs were used to interpret the results, which were triangulated with the extant literature.

## Results

### Quantitative results

**Fig 1:**
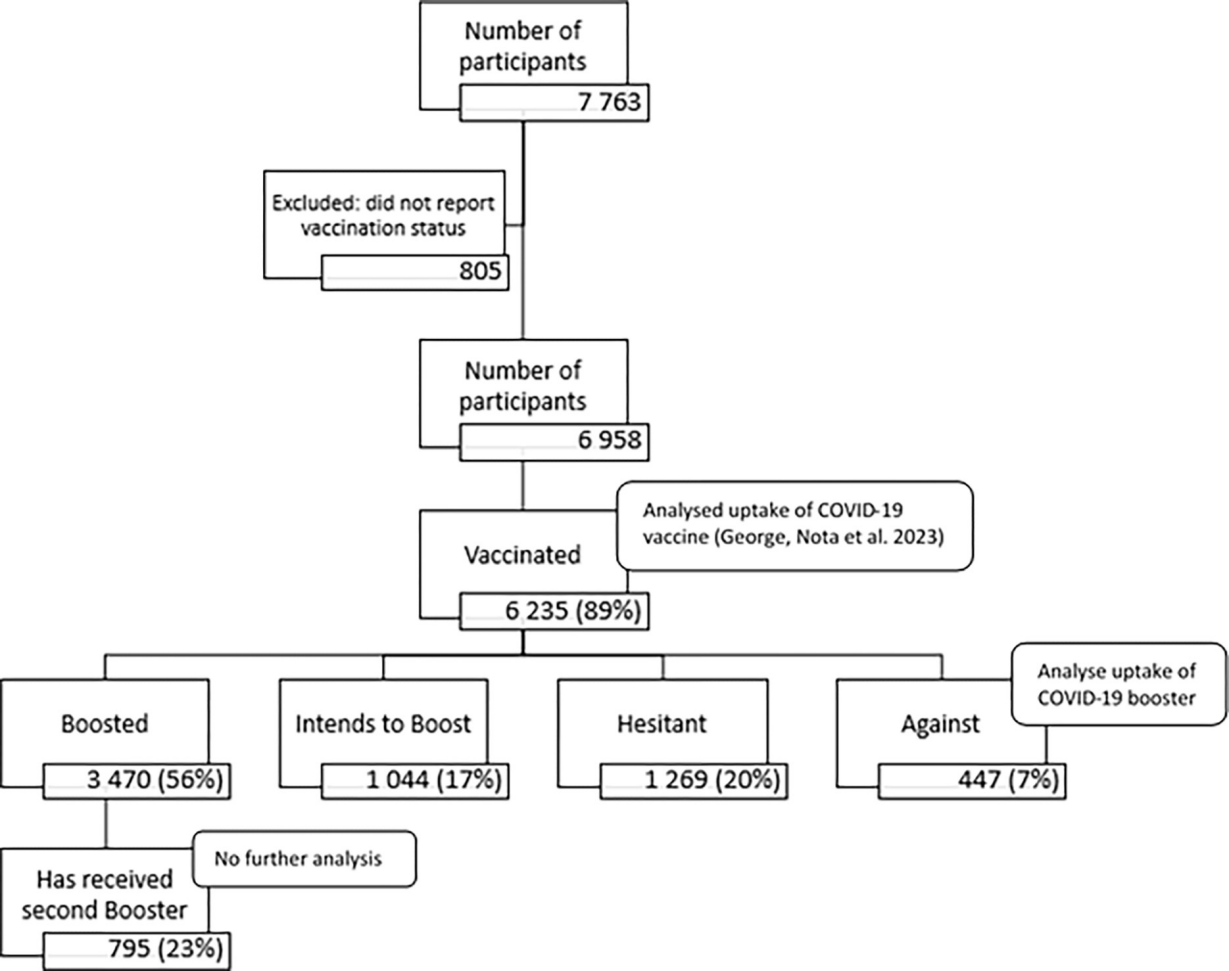
Flow diagram of COVID-19 vaccination and booster behaviour in our sample.

There were 7 763 participants in the study. Of these, 6 235 participants were vaccinated, with 3 470 (56%) having received at least one booster dose. See Fig 1 for a breakdown of vaccination and booster dose behaviour of the study participants.

Table 1 presents the univariate logistic regression analysis consisting of our first outcome variable and demographic measures. The older HCW cohorts, those aged 35 to 49 years (OR = 1.30 [95% CI: 1.15–1.46]), and those aged 50 years or older (OR = 2.66 [95% CI: 2.32–3.05]) were more likely to have received the booster dose than HCWs in the youngest cohort (Table 1). Females were less likely than males to have received the booster dose (OR = 0.88 [95% CI: 0.79–0.98]) while Coloured (OR = 1.51 [95% CI: 1.23–1.85]), Indian (OR = 1.32 [95% CI: 1.07–1.63]) and White (OR = 2.15 [95% CI: 1.91–2.43]) HCWs were more likely to have received the booster dose than their Black African counterparts. Non-South African HCWs were less likely to have received the booster dose (OR = 0.81 [95% CI: 0.69–0.96]) than South African HCWs. HCWs who had a chronic condition were also more likely to have received the booster dose (OR = 1.19 [95% CI: 1.07–1.32]) than those who did not have a chronic condition.

**Table 1:** Booster behaviour according to demographics.

Measures	Received Booster (n = 3 470) (55.70%)	No Booster (base case) (n = 2 760) (44.30%)	Odds Ratio^1^ [95% CI]
**What is your age?**			
Younger than 35 years old	899 (46.51%)	1 034 (53.49%)	1.00
35 to 49 years old	1 362 (53.10%)	1 203 (46.90%)	1.30 [1.15–1.46]
50 years old or older	1 201 (69.87%)	518 (30.13%)	2.66 [2.32–3.05]
**What is your gender?**			
Male	1 084 (57.72%)	794 (42.28%)	1.00
Female	2 363 (54.78%)	1 951 (45.22%)	0.88 [0.79–0.98]
**What is your race?**			
African	1 739 (49.15%)	1 799 (50.85%)	1.00
Coloured	265 (59.42%)	181 (40.58%)	1.51 [1.23–1.85]
Indian	224 (56.14%)	175 (43.86%)	1.32 [1.07–1.63]
White	1 143 (67.59%)	548 (32.41%)	2.15 [1.91–2.43]
**What is your nationality?**			
South African	3 083 (56.39%)	2 384 (43.61%)	1.00
Non-South African	344 (51.42%)	325 (48.58%)	0.81 [0.69–0.96]
**Do you have any chronic conditions?** ^**2**^			
No	2 169 (54.16%)	1 836 (45.84%)	1.00
Yes	1 301 (58.50%)	923 (41.50%)	1.19 [1.07–1.32]
**What is your professional role?**			
Nurse	1 333 (57.06%)	1 003 (42.94%)	1.00
Doctor	710 (67.75%)	338 (32.25%)	1.58 [1.35–1.84]
All other^3^	919 (52.01%)	848 (47.99%)	0.81 [0.72–0.92]
**Are you in direct contact with patients?**			
No	385 (54.00%)	328 (46.00%)	1.00
Yes	2,564 (58.05%)	1,853 (41.95%)	1.17 [1.00–1.38]
**In which health sector do you work?**			
Public	1 257 (59.49%)	856 (40.51%)	1.00
Private	1 052 (58.15%)	757 (41.85%)	0.94 [0.83–1.07]
NGO	266 (51.55%)	250 (48.45%)	0.72 [0.59–0.87]
Public and private	230 (51.00%)	221 (49.00%)	0.70 [0.57–0.86]
Other	157 (59.70%)	106 (40.30%)	1.00 [0.77–1.30]
**How many years have you worked for?**			
Less than 5 years	348 (48.27%)	373 (51.73%)	1.00
5 to 9 years	566 (48.75%)	595 (51.25%)	1.01 [0.84–1.22]
10 to more years	2 046 (62.61%)	1 222 (37.39%)	1.79 [1.52–2.11]

^1^Univariate Logistic Regression

^2^Chronic conditions were defined as having one or more of the following: diabetes, hypertension, respiratory disease, HIV, and other chronic diseases

^3^This category consists of Pharmacist, Allied health professional, Personal service worker, Paramedic, other health professional, and other.

https://figshare.com/articles/figure/Table_1/24486160

Doctors were more likely to have received the booster dose than nurses (OR = 1.58 [95% CI: 1.35–1.84]) while all other HCWs (such as Pharmacists, Allied health professionals, Personal service workers, and Paramedics) were less likely to have received the booster dose when compared to nurses (OR = 0.81 [95% CI: 0.72–0.92]). HCWs who were in direct contact with patients were more likely to have received the booster dose than those not in direct contact with patients (OR = 1.17 [95% CI: 1.00–1.38]). HCWs who had worked for 10 or more years were more likely to have received the booster dose (OR = 1.79 [95% CI: 1.52–2.11]) than their less experienced colleagues.

In Table 2, we conducted univariate logistic regression analysis with our first outcome variable and several COVID-19 and vaccine related measures. HCWs who reported experiencing an adverse reaction following initial vaccination were less likely to have received the booster dose than those who had not experienced any side-effects (OR = 0.88 [95% CI: 0.80–0.98]). Adverse reaction was defined as experiencing any one of the following: low/high fever, pain/swelling at the injection site, dyspnoea, malaise, or any reaction that required hospitalisation. HCWs who had felt obligated to get vaccinated, either for occupational reasons, mandatory by employer, for travelling purposes or pressured by family and friends, were also less likely to have received the booster dose (OR = 0.54 [95% CI: 0.48–0.59]). HCWs who had previously received a flu vaccine (OR = 1.99 [95% CI: 1.56–2.55]) and those who planned to get vaccinated against other diseases (OR = 1.98 [95% CI: 1.55–2.53]) were more likely to have received the booster dose.

**Table 2:** Booster behaviour according to COVID-19 and vaccine related measures.

Measures	Received Booster (n = 3 470) (55.70%)	No Booster (base case) (n = 2 760) (44.30%)	Odds Ratio^4^ [95% CI]
**Have you ever tested positive for COVID?**			
No	1 722 (55.44%)	1 384 (44.56%)	1.00
Yes	1 748 (55.97%)	1 375 (44.03%)	1.02 [0.92–1.12]
**Do you perceive yourself as a risk to your patients?** ^**5**^			
No	112 (58.95%)	78 (41.05%)	1.00
Yes	392 (59.13%)	271 (40.87%)	1.00 [0.72–1.39]
**Do you perceive your patients as a risk to you?** ^**5**^			
No	109 (58.29%)	78 (41.71%)	1.00
Yes	395 (59.22%)	272 (40.78%)	1.03 [0.74–1.44]
**Did you have a bad reaction when you were vaccinated?**			
No	1 537 (57.39%)	1 141 (42.61%)	1.00
Yes	1 932 (54.47%)	1 615 (45.53%)	0.88 [0.80–0.98]
**Did you feel obligated to get vaccinated?**			
No	2,192 (62.22%)	1,331 (37.78%)	1.00
Yes	1,274 (47.13%)	1,429 (52.87%)	0.54 [0.48–0.59]
**Have you received a flu vaccination?**			
No	165 (43.77%)	212 (56.23%)	1.00
Yes	537 (60.88%)	345 (39.12%)	1.99 [1.56–2.55]
**Do you plan to get vaccinated against other diseases?**			
No	162 (43.78%)	208 (56.22%)	1.00
Yes	540 (60.74%)	349 (39.26%)	1.98 [1.55–2.53]

^4^Univariate Logistic Regression

^5^Direct patient contact only.

https://figshare.com/articles/figure/Table_2/24486202

In the following tables, we conducted univariate logistic regression analysis with our second outcome variable and our demographic measures (Table 3), and several COVID-19 and vaccination measures (Table 4). HCWs who intended to get the booster dose were grouped with HCWs who had already received the booster dose, and compared with HCWs who were hesitant or against the booster doses, with similar results emerging from these analyses when comparing HCWs had have received the booster dose with those that hadn’t. There were two exceptions. Firstly, non-South African HCWs were now more likely to have received or intended to receive the booster (OR = 1.28 [95% CI: 1.06–1.55]) than their South African counterparts. Secondly, there was no significant difference between those who were boosted or intended to get the booster and those who were hesitant or against booster doses across the health care sectors.

**Table 3:** Booster behaviour, intentions, hesitancy and reluctance according to demographics.

Measures	Received and Intend (n = 4 514) (72.46%)	Hesitant and Against (base case) (n = 1 716) (27.54%)	Odds Ratio^6^ [95% CI]
**What is your age?**			
Younger than 35 years old	1 289 (66.68%)	644 (33.32%)	1.00
35 to 49 years old	1 826 (71.19%)	739 (28.81%)	1.23 [1.08–1.40]
50 years old or older	1 391 (80.92%)	328 (19.08%)	2.11 [1.81–2.46]
**What is your gender?**			
Male	1 432 (76.25%)	446 (23.75%)	1.00
Female	3 054 (70.79%)	1260 (29.21%)	0.75 [0.66–0.85]
**What is your race?**			
African	2 349 (66.39%)	1 189 (33.61%)	1.00
Coloured	324 (72.65%)	122 (27.35%)	1.34 [1.07–1.67]
Indian	294 (73.68%)	105 (26.32%)	1.41 [1.12–1.79]
White	1 420 (83.97%)	271 (16.03%)	2.65 [2.28–3.07]
**What is your nationality?**			
South African	3 930 (71.89%)	1 537 (28.11%)	1.00
Non-South African	513 (76.68%)	156 (23.32%)	1.28 [1.06–1.55]
**Do you have any chronic conditions?** ^**7**^			
No	2 833 (70.74%)	1 172 (29.26%)	1.00
Yes	1 681 (75.58%)	543 (24.42%)	1.28 [1.13–1.44]
**What is your professional role?**			
Nurse	1 684 (72.09%)	652 (27.91%)	1.00
Doctor	864 (82.44%)	184 (17.56%)	1.81 [1.51–2.18]
All other^8^	1 223 (69.21%)	544 (30.79%)	0.87 [0.76–0.99]
**Are you in direct contact with patients?**			
No	494 (69.28%)	219 (30.72%)	1.00
Yes	3 261 (73.83%)	1156 (26.17%)	1.25 [1.05–1.48]
**In which health sector do you work?**			
Public	1 552 (73.45%)	561 (26.55%)	1.00
Private	1 329 (73.47%)	480 (26.53%)	1.00 [0.86–1.15]
NGO	391 (75.78%)	125 (24.22%)	1.13 [0.90–1.41]
Public and private	313 (69.40%)	138 (30.60%)	0.81 [0.65–1.02]
Other	187 (71.10%)	76 (28.90%)	0.88 [0.66–1.18]
**How many years have you worked for?**			
Less than 5 years	494 (68.52%)	227 (31.48%)	1.00
5 to 9 years	819 (70.54%)	342 (29.46%)	1.10 [0.89–1.34]
10 to more years	2 457 (75.18%)	811 (24.82%)	1.39 [1.16–1.66]

^6^Univariate Logistic Regression

^7^Chronic conditions were defined as having one or more of the following: diabetes, hypertension, respiratory disease, HIV, and other chronic diseases

^8^This category consists of Pharmacist, Allied health professional, Personal service worker, Paramedic, other health professional, and other.

https://figshare.com/articles/figure/Table_3/24486211

**Table 4:** Booster behaviour, intentions, hesitancy, and reluctance according to COVID-19 and vaccine related measures.

Measures	Received and Intend (n = 4 514) (72.46%)	Hesitant and Against (base case) (n = 1 716) (27.54%)	Odds Ratio^9^ [95% CI]
**Have you ever tested positive for COVID?**			
No	2 276 (73.28%)	830 (26.72%)	1.00
Yes	2 237 (71.63%)	886 (28.37%)	0.92 [0.82–1.02]
**Do you perceive yourself as a risk to your patients?** ^**10**^			
No	137 (72.11%)	53 (27.89%)	1.00
Yes	507 (76.47%)	156 (23.53%)	1.25 [0.87–1.80]
**Do you perceive your patients as a risk to you?** ^**10**^			
No	136 (72.73%)	51 (27.27%)	1.00
Yes	508 (76.16%)	159 (23.84%)	1.19 [0.82–1.73]
**Did you have a bad reaction when you were vaccinated?**			
No	2 018 (75.35%)	660 (24.65%)	1.00
Yes	2 495 (70.34%)	1 052 (29.66%)	0.77 [0.69–0.86]
**Did you feel obligated to get vaccinated?**			
No	2 822 (80.10%)	701 (19.90%)	1.00
Yes	1 688 (62.45%)	1 015 (37.55%)	0.41 [0.36–0.46]
**Have you received a flu vaccination?**			
No	234 (62.07%)	143 (37.93%)	1.00
Yes	687 (77.89%)	195 (22.11%)	2.15 [1.65–2.79]
**Do you plan to get vaccinated against any other diseases?**			
No	205 (55.41%)	165 (44.59%)	1.00
Yes	716 (80.54%)	173 (19.46%)	3.33 [2.55–4.33]

^9^Univariate Logistic Regression

^10^Direct patient contact only.

https://figshare.com/articles/figure/Table_4/24486229

Table 5 reveals the reasons HCWs listed for why they hadn’t received their booster doses. Of all the available options, the majority of both males (22.94%) and females (22.93%) believed that the initial COVID-19 vaccine dose offered sufficient protection. The second most documented reason for females centred on concerns about possible complications in the future (21%) while for males, 19.12% did not believe that the booster dose was effective.

**Table 5:** Barriers to boost.

	n = 1 716 (%)	Male (%)	Female (%)
Why do you not want to take the booster dose?			
I don’t believe the booster is effective.	164 (13.83)	19.12	11.70
I had adverse effects after vaccination and I fear it will be worse after the booster.	162 (13.66)	8.24	15.84
I believe that the vaccine gives me sufficient protection.	275 (22.93)	22.94	22.93
A friend/family member had severe adverse effects after vaccination.	38 (3.20)	1.76	3.78
I have logistical difficulties (transport to the facility, time etc).	11 (0.93)	1.76	0.59
I am concerned about possible complications in the future.	233 (19.48)	15.59	21.04
I’ve had COVID-19 and I believe it gives me sufficient protection in addition to the vaccine.	133 (11.13)	12.06	10.76
Other	176 (14.84)	18.53	13.36
No reason given^11^	524		

^11^Not included in percentage calculation.

https://figshare.com/articles/figure/Table_5/24486256

The data further revealed that 15.84% of females had not received their booster dose because they had experienced adverse effects after initial vaccination, compared to 8.24% of males who listed this as a reason.

### Qualitative results

We identified four themes regarding HCWs’ uptake and experiences of COVID-19 vaccine boosters: *(1) Vaccination as a routine practice among HCWs; (2) Emergence of new COVID-19 variants necessitating vaccine boosters; (3) Fear of potential side-effects from COVID-19 vaccine boosters; and (4) Limited value of COVID-19 vaccine boosters*. Overall, participants held positive views towards COVID-19 vaccine boosters, with most highlighting the importance of boosters in maintaining population immunity. However, some HCWs remained both hesitant and against getting the booster, questioning its value, while raising safety concerns, which largely emanated from personal and observed experiences of adverse events following initial vaccination or after receiving a booster dose. These are discussed more fully below.

#### 1. Vaccination as a routine practice among HCWs

Participants recognized the value of regular vaccination, often citing that the influenza vaccine was part of their regular health routine. These participants were easily able to accept the importance of regular COVID-19 vaccine boosters, both to provide ongoing protection against COVID-19 disease and severe illness, and for protecting against potential virus variants or mutations.

*I actually said to my family*, *this is going to end up being like a flu vaccine*. *It’s something that we’re going to have to get annually*, *like we all get our flu vaccines annually*. *So for me it was never a question of maybe getting only one or two vaccines*, *and then it’s going to stop*. *This is a virus*, *and it changes*, *and it mutates*, *and we have to try and keep up with it*.

(P20, Nurse, F, Vaccinated)

Another participant went on to explain:

*Personally*, *I think it is important*. *I think that again I’m a believer*, *so I have taken flu vaccine annually*, *and so if I believe that you know I don’t take a flu vaccine once and expect it to cover me for the rest of my life* […] *I think boosters are important in terms of coverage*, *to be fully vaccinated*.

(P18, Other HCW, F, Vaccinated)

HCWs continue to feel susceptible to infections, with older HCWs and those with chronic diseases feeling particularly vulnerable to COVID-19.

*I’ve been boosted yes…Because it’s a virus so that’s what this virus job is to infiltrate and cause chaos*, *so you need to be one step ahead*, *just like the flu*, *so you need to get boosted*, *especially my age*, *I’m over 50*.

(P11, Other HCW, Female, Vaccinated)

#### 2. Emergence of new COVID-19 variants necessitating vaccine boosters

Participants recognised the limited effect vaccines had on the new emerging variants, relying therefore on boosters to provide protection against virus mutations.

*I’ve had the booster*. *I think it is important because of the variants [*…] *it’s changing*.

(P9, Nurse, F, Vaccinated)

Participants felt that the booster dose improved individual’s immune response.

*I did get a booster*. *I think you know*, *it’s probably quite important just to help your immune system to create memory cells adequately*, *and to make sure that you know*, *you’re actually able to bolster an adequate response*.

(P19, Doctor, F, Vaccinated)

#### 3. Fear of potential side-effects from COVID-19 vaccine boosters

Some HCWs experienced adverse side-effects following their initial vaccination. This, coupled with observing colleagues and others suffering from side-effects after receiving the vaccine or booster shots, dissuaded them from returning for their booster doses.

*There were many stories about the vaccine and one ended up not getting the booster* […] *I was concerned that maybe one would have side effects and getting a booster and I thought*, *maybe I also would have side effects as well*.

(P10, Nurse, Female, Vaccinated)

Another HCW added:

*Most of my colleagues have not yet gone for the booster but I think most of them had…a few of them still have that fear of*, *like I mentioned experiencing severe side effects of the vaccine* […]

*I think*, *with the booster dose*, *I delayed it due to the side effects that I had with the second dose of the vaccine*.

(P28, Nurse, F, Vaccinated)

In addition to the side-effects, participants raised concerned around the safety of booster doses for those that were pregnant.

*The few colleagues that I spoke to*, *they said to me that they felt so bad after the second vaccination*, *they’re not going for the boosters*. *And I also have a colleague now that’s pregnant and she’s not sure what the effects might be on the foetus*.

(P3, Other HCW, M, Unvaccinated)

#### 4. Limited value of COVID-19 vaccine boosters

Some participants questioned the value of additional booster doses, suggesting that the initial vaccine provided adequate protection against infection and severe illness.

*I had COVID*, *I think*, *last year December*, *and it was not bad*, *so I don’t think the booster is going to make any difference*, *because the symptoms were not bad*, *so I’m not doing it*.

(P21, Other HCW, F, Vaccinated)

Some participants did not feel particularly vulnerable to COVID-19 infection, suggesting that their risk profile did not warrant getting booster doses.

*I’ve not gone on to receive the booster dosages*. *I personally*, *for my own personal risk perspective*, *I didn’t think it was that important*. *I don’t have particularly high-risk factors for severe COVID-19*, *and I didn’t feel that it was necessary for me personally*.

(P12, Other HCW, Female, Vaccinated)

Even among unvaccinated participants, getting the initial vaccine and the booster dose was often expressed as unnecessary.

*I would opt for my natural response*… *you know development of antibodies etcetera*, *and see what will happen*. *Actually*, *my option was natural response versus COVID-19 vaccines and boosters*.

(P2, Nurse, M, Unvaccinated)

## Discussion

In our study, 56% of HCWs had received their initial COVID-19 booster vaccination while a further 17% reported their intention to get the booster dose within 12 months of completing the survey. Of those that had received their first booster dose, 23% had already received their second at the time of the study. These percentages are higher than those observed among the general South African public, where only approximately 24% had received a vaccine booster by March 2023 [16]. These percentages are slightly lower than those reported in studies on HCWs undertaken in high income countries [7, 9]. However, it should be noted that high income countries have had access to vaccines for a longer period.

Previously published data on primary COVID-19 vaccination among the same study sample highlighted significant disparities in vaccine uptake by age, gender, race, job roles and presence or absence of a chronic health condition [4]. This study redemonstrated some of these disparities with COVID-19 booster vaccination uptake rates correlating to age, some job roles (other HCWs) and those who had a chronic condition. This was consistent with other studies which revealed that older groups and HCWs with a chronic condition were more likely to get their booster doses [17–19].

There were, however, some notable incongruities with respect to findings from initial vaccine uptake behaviour [4], with females showing higher rates of hesitancy towards getting booster doses than their male colleagues, and doctors more willing than nurses to receive boosters. Our data suggest that a higher proportion of females experienced adverse side effects following initial vaccination, and this, in part, fuelled hesitancy towards boosters, together with greater concern for potential future complications. Fear of post-vaccination side effects has been identified as one of the key barriers for accepting COVID-19 vaccine booster doses in related literature [20]. Our qualitative data provided further elaboration, with HCWs not only experiencing perceived adverse side-effects following their initial vaccination, but also observing both colleagues and patients suffering side-effects which elevated the level of fear and subsequently fuelled hesitancy towards booster doses.

Two further dominant reasons for not getting the booster doses–for both males and females–centred around the belief that the initial vaccination provided adequate protection and that subsequent boosters were unnecessary or ineffective. The IDIs revealed contrasting perspectives, with some believing that boosters were necessary in combating the new virus strains whereas others questioned the efficacy of available vaccine boosters. HCWs operating in other contexts also reported that boosters would be required to counter new emerging strains of the virus [8].

This study also found disparities in COVID-19 booster vaccination uptake among HCWs who were patient facing, which can be viewed as a proxy for increased perceived risk and fear of transmitting to patients, both factors correlating with increased uptake of boosters in another study [18]. HCWs who had historically received annual influenza vaccinations, along with colleagues who intended getting vaccinated for other diseases also demonstrated higher probability of having received booster doses. The qualitative data reveals that getting annual influenza vaccination is a routine practice amongst some HCWs, who realise the value of vaccination boosters more broadly than just for COVID-19, as a counter measure against rapidly mutating viruses. Effecting optimal vaccination behaviour amongst HCWs has proven challenging, with studies revealing that attitudes, self-efficacy, perceived risk/benefit, cues to action, and social norms as key constructs which correlated with uptake rates [21]. Literature reviewing predictors of vaccination hesitancy more broadly, has suggested that any interventions aimed at improving uptake rates should be guided by behaviour change theories [21], with tailored education and risk messaging having already proven successful in certain contexts [7]. Our earlier study [4] also suggested that better education regarding scientific and safety/oversight issues in vaccine development was needed to offset perceptions that vaccines were rushed to implementation without what was considered to be the conventional reviews and measures regarding safety and efficacy.

Vaccination booster rates, along with the intention to get the booster dose, remain high among HCWs in South Africa. However, not all HCWs who had vaccinated were committed to receiving boosters. Enthusiasm for further boosters appears to be waning as indicated by the low percentage (23%) who had received their second booster, even though most would have been eligible. The slowing uptake of boosters is evident not only among the South African general population [16] but among populations globally [22]. This is of concern within a context of potential future variants and other infectious disease epidemics in general. The uptake of vaccines and subsequent boosters are also likely to be influenced by the spike in population infection and subsequent increase in the morbidity and mortality rates, however, these alerts from both government officials and the media have remained subdued. Reports suggest that people are avoiding media coverage on COVID-19 which is in turn reducing the demand for and subsequent generation of news on COVID-19 [23]. With less global attention being given to COVID-19, vaccination and booster fatigue may be setting in. Arresting this slowdown in the uptake of COVID-19 vaccines and boosters will require a reinvigorated communication strategy–guided by behavioural theories, and evidence based public education, as has been previously suggested. This, however, does not appear imminent, with the result that South Africa (and potentially other countries) is overstocked with vaccines which are on the verge of expiry and facing the prospect of being destroyed [24].

### Strengths and limitations

Our study adds to the limited available data regarding COVID-19 booster vaccination uptake among HCWs, especially data available from Africa. Findings are expected to guide future vaccine campaigns and public health strategies to build vaccine confidence among HCWs and the general population.

The study is limited by using an unrestricted self-administered survey that was dependent on the online reachability of HCWs on selected databases. Limitations in study design may have introduced selection bias and may have limited generalizability amongst HCWs in South Africa.

## Conclusion

This is the first study examining the uptake of vaccine booster doses among South African HCWs, offering valuable insights into drivers of hesitancy, revealing the perspectives of HCWs, all of which will hopefully influence the design of future vaccination programmes. This study shows that key demographics influence uptake of boosters, as was the case with primary vaccine doses. Age, race, gender, job roles and chronic health conditions were all factors contributing to the uptake of vaccine booster doses. Booster rates in our sample of HCWs were high, with the majority having had or intending to get their booster doses. The data revealed that females were concerned about the potential side effects and future complications which may arise from booster doses. Hesitancy was also fuelled by those who questioned the value of boosters relative to the protection afforded to them from their primary vaccination, or whether these boosters were effective against the emerging virus strains. Efforts are required to ensure that vaccination against disease becomes normative practice amongst HCWs, with this study showing participants who had historically taken the influenza vaccine more willing to get the COVID-19 booster doses. These normative practices can only be inculcated amongst HCWs through effective evidence-based training and communication strategies.

## Supporting information

S1 Text Inclusivity in global research. (DOCX)
